# Olean (1,7-dioxaspiro[5.5]undecane): A Novel Intraspecific Chemical Cue in *Coraebus undatus* (F.) (Coleoptera: Buprestidae)

**DOI:** 10.3390/insects12121085

**Published:** 2021-12-03

**Authors:** Sergio López, José María Álvarez-Calero, Josep Maria Riba-Flinch, María Milagro Coca-Abia, Antoni Torrell, Carmen Quero

**Affiliations:** 1 Department of Biological Chemistry, Institute for Advanced Chemistry of Catalonia (IQAC-CSIC), Jordi Girona 18-26, 08034 Barcelona, Spain; jacqbm@cid.csic.es; 2 Plant Pathologist and Consulting Arborist, 17320 Tossa de Mar, Spain; jmriba2001@gmail.com; 3 Centro de Investigación y Tecnología Agroalimentaria de Aragón (CITA), Instituto Agroalimentario de Aragón (Universidad de Zaragoza) (IA2), Avda, Montañana 930, 50059 Zaragoza, Spain; mcoca@aragon.es; 4 Forestal Catalana-Departament d’Acció Climàtica, Alimentació i Agenda Rural, Carrer del Dr. Roux, 80, 08017 Barcelona, Spain; atorrells@gencat.cat

**Keywords:** *Coraebus undatus*, Buprestidae, oak pest, 1,7-dioxaspiro[5.5]undecane (olean), chemical ecology, behavior, purple prism trap

## Abstract

**Simple Summary:**

The flathead oak borer *Coraebus undatus* (F.) (Coleoptera: Buprestidae) is a major pest of cork oak (*Quercus suber*) along the Mediterranean Basin that generates significant economic losses in the cork industry. Larvae bore long galleries and feed on the cork generating layer, thus affecting its quality. At present, the semiochemistry of this species is poorly known, and therefore the elucidation of chemicals involved in its intraspecific communication may allow the development of novel control tools. We determined that both sexes release the compound 1,7-dioxaspiro[5.5]undecane, and the biological activity of the compound was addressed by means of electroantennography and behavioral assays. The attractiveness of the compound on both sexes under laboratory conditions contrasts to its performance in field trials, which may be explained by features inherent to the methodological design (e.g., the absence of a contextually related visual stimulus or trap deployment height). This is the first time in which an intraspecific compound has been reported as attractive for the species, and practical implications for the assessment of its activity under natural conditions are also further discussed.

**Abstract:**

The main aim of this work was to identify semiochemicals from the jewel beetle *Coraebus undatus* (F.) (Coleoptera: Buprestidae) that may aid in the improvement of current monitoring tools. First, HS-SPME collections revealed that individually sampled adults (>7 days old) of both sexes release the spiroacetal 1,7-dioxaspiro[5.5]undecane (olean). Electroantennographic recordings from both sexes exposed to increasing amounts of olean followed a dose-dependent pattern, with females being more responsive than males to the highest amount of the compound (100 µg). In double-choice assays, adults older than seven days were significantly attracted to olean, whereas this attraction was not detected in insects aged less than seven days. Indeed, a repellent effect was observed in young females. Subsequent field trials employing sticky purple prism traps revealed that there were no differences among the number of insects caught in control and olean-baited traps at two different release rates (0.75 and 3.75 mg/day). Interestingly, all the trapped specimens were determined as mated females, regardless of the presence of olean. Overall, these findings provide a basis for unraveling the chemical ecology of the species, although further research is still needed to determine the specific role of this compound within the chemical communication of the species.

## 1. Introduction

Insect pests are recognized as significant drivers of forest ecosystems’ disturbance worldwide, in addition to other biotic and abiotic factors [1]. Among the plethora of harmful insect taxa, several bark and wood-boring beetles represent a serious threat for the forest health and economic exploitation of forest resources, not only due to the direct damage they cause [2,3], but also for the pathogens they may transmit as vector insects [4,5]. Jewel beetles (Coleoptera: Buprestidae) are included within these damaging organisms [3]. Most buprestid species are saproxylic, in which their progeny develops under the bark of decayed and dead trees. However, some species colonize living stressed and weakened trees [6,7,8,9], causing the death of the host, due to the direct feeding activity of the larvae. One of the most destructive species is the emerald ash borer *Agrilus planipennis* Fairmaire, an Asian invasive species that causes serious damage to ash species (*Fraxinus* spp.) native to North America. Since its first detection in Michigan and Ontario in 2002 [10], *A. planipennis* has caused the mortality of hundreds of millions of ash trees, leading to losses of billions of dollars in the United States and Canada in terms of preventive measures and management [11,12]. Under this scenario, vast research has been devoted during the last two decades to develop efficient management strategies against this destructive species, with relevant works focused on its chemical ecology. In this regard, it has been elucidated that some host bark sesquiterpenes, the host green leaf volatile (*Z*)-3-hexen-1-ol, and a female-released pheromone, namely (*Z*)-3-dodecen-12-olide, are involved in the attraction of males and females to sticky green and purple prism traps [comprehensively reviewed in [13]], with each sex displaying a marked preference for a concrete visual-olfactory cue combination.

To the best of our knowledge, fifteen native jewel beetle species are regarded as damaging agents in different areas of Europe, especially affecting broadleaf trees [3]. In the Mediterranean Basin, the flathead oak borer (FOB) *Coraebus undatus* (F.) is considered as a primary pest species that attacks apparently healthy cork oaks (*Quercus suber* L.) [3], and even though it does not directly kill the tree, severe infestations may weaken the host and favor the incidence of more noxious organisms [14]. The species shows a biennial life-cycle [15,16], although it has been suggested that it may complete the larval development in a single year [17]. Females lay eggs in bark crevices from May to September, and after hatching, larvae burrow into the cork and build long (up to 2 m) and wide (3–5 mm) S-shaped vertical galleries in the cork-generating layer [3]. As a consequence, cork-producing tissues are damaged and the quality of the harvested material drastically decreases, leading to severe economic losses for the cork industry. For instance, in the region of Extremadura (Spain), the annual economic loss in cork production attributable to *C. undatus* has been estimated to be EUR 5 million [14].

Currently, no effective management measure against the FOB has been implemented, and only silvicultural practices, such as removing the bark from the affected oak, are useful for detecting the presence of larvae underneath [14]. Therefore, the development of novel efficient tools for surveillance and/or mass trapping of dispersing adults is urgently needed. At present, the sole monitoring tool can be described as a blend comprising five host green leaf volatiles in combination with sticky purple prism traps [18]. This trapping approach was demonstrated to be more effective than the use of other lures (ethanol and a blend of nonanal, decanal, and geranylacetone), regardless of the trap employed (purple prism or Lindgren multiple-funnel trap). Interestingly, these host volatiles have been proven to be attractive for the congeneric species *Coraebus florentinus* (Herbst) under laboratory conditions [19]. Nonetheless, little is still known about the intraspecific or insect-host communication in *C. undatus*, and consequently the elucidation of novel semiochemicals would represent an alternative approach for monitoring and/or controlling the species.

In this study we focused on the volatile profile of both sexes of the FOB, in order to identify potential compounds that may be relevant in the intraspecific communication of the species. To achieve our aims, we (1) collected volatiles emitted by adult beetles of both sexes by headspace solid-phase microextraction, (2) assessed the electroantennographic and behavioral response of both sexes to 1,7-dioxaspiro[5.5]undecane (hereafter referred as olean), and, finally, (3) evaluated the attractiveness of the compound in a cork oak stand. These findings represent a preliminary basis for unraveling the chemical ecology of the species, which in turn may contribute to the improvement of current surveillance strategies.

## 2. Materials and Methods

### 2.1. Insects

Newly emerged *C. undatus* adults were directly collected from infested *Q. suber* trees located in the provinces of Barcelona and Girona (NE Spain) during June and July 2021. For beetle collection, trunk sections with external symptoms of infestation were covered with a mosquito net tightly fastened with foam stripes at the top and bottom, and the headspace between the net and the trunk was daily checked for the collection of emerged adult FOB. When a new adult was found between the net and the trunk, a small cut was made with a jackknife to pick up the insect, and afterwards the slit was sealed with staples. These emerged adults were individually placed into 2 mL Eppendorf^®^ tubes, and immediately taken to the facilities of the Institute for Advanced Chemistry of Catalonia. For insect maintenance, they were individually kept at 25 ± 1 °C, 55 ± 5% RH and 16:8 L:D photoperiod inside PET plastic cups (720 mL, 114 mm height, 85 mm O.D., Entomopraxis, Barcelona, Spain) covered with a mesh lid. As a feeding source, each cup contained fresh *Q. suber* twigs inserted into a capped plastic container (40 mm height, 43 mm O.D.) with a 7 mm hole drilled in the cap to hold the twigs, and filled with water (30 mL) to prevent dehydration. The replacement of twigs and water was undertaken every two days. Sex of live beetles was determined according to the morphology of the 8–9th sternites [20], and once dead, by removal and inspection of the genitalia in order to corroborate the preliminary sex assignation.

### 2.2. Chemicals

Racemic olean (98%) was purchased from Alfa-Aesar (Haverhill, MA, USA). For analytical procedures and solutions, n-hexane of GC purity (SupraSolv^®^, Merck, Darmstadt, Germany) was used as solvent.

### 2.3. Headspace Solid-Phase Microextraction (HS-SPME)

Each volatile collection consisted of one individual (>7 days old) placed in a 15 mL clear glass vial sealed with a screw cap with a PFTE/silicone septum, and exposed to a polydimethylsiloxane/divinylbenzene-coated fiber (PDMS/DVB, 65 µm; Supelco, Merck-Sigma Aldrich, Madrid, Spain) for 4 h. A total of *n* = 8 collections with different individuals in each sampling were conducted per sex. Before the first time of use, the solid-phase microextraction (SPME) fiber was thermally cleaned for 0.5 h at 250 °C in the injection port of a gas chromatograph. All the collections were conducted under artificial light at room temperature, from 10:00 a.m. to 18:00 p.m.

### 2.4. Chemical Analysis

After volatile collection, HS-SPME samples were immediately analyzed by gas chromatography coupled to mass spectrometry, by injection in splitless mode into a Thermo Finnigan Trace 2000 GC system coupled to a Trace MS quadrupole mass spectrometer (Thermo Fisher Scientific, Madrid, Spain). Helium (1 mL/min) was the carrier gas, and the column used was a TR-5MS (30 m × 0.25 mm I.D. × 0.25 µm; Thermo Fisher Scientific), with the following temperature program: 40 °C (held for 5 min) to 150 °C at 5 °C/min and increased to 310 °C at 10 °C/min (held for 10 min). The MS was used in the electron impact mode at 70 eV. The mass range scanned was 40–500, at 1.0 scan/s. Olean was identified by comparison of its mass spectrum with those of a synthetic standard and a commercial library (NIST Registry of Mass Spectral Data, 2005; Wiley, 2000).

### 2.5. Electroantennographic (EAG) Response

To evaluate the EAG dose response of both sexes of the FOB (>7 days old, males *n* = 5; females *n* = 10) to olean (1, 10, and 100 µg), a standardized procedure was followed for sample preparation [19]. Briefly, one antenna of each adult was excised, and the last antennomeres removed with a microscalpel and fixed to a forked microelectrode holder (Syntech, Kirchzarten, Germany) with a drop of conductive gel (Spectra 360, Parker Lab. Inc., Hellendoorn, The Netherlands). The tip of the antenna was attached to the recording microelectrode, and the proximal part was fixed to the reference microelectrode. The holder was then connected to an EAG Combi-Probe (Syntech) coupled to a MP-5 micromanipulator (Syntech). The antennal preparation was subjected to a continuous humidified pure air flow (ca. 650 mL/min) delivered through the main branch of a glass tube (7 cm long × 5 mm diameter) placed 1 cm over the sample. Olean-delivering air stimuli were carried out by giving air puffs (ca. 300 mL/min) for 100 ms through 150 mm long disposable glass Pasteur pipettes with the aid of a stimulus controller CS-01 (Syntech). Each pipette contained a Whatman filter paper disc (2.5 cm diameter, Merck-Sigma Aldrich) onto which the corresponding olean quantity had been loaded. These amounts were obtained by loading 10 µL of 0.1, 1.0, or 10 µg/µL olean dilutions (in hexane). Two puffs per olean amount were applied to each antennal preparation in increasing order of concentration, with an interval of 60 s between puffs. Control puffs with the hexane alone (solvent) were intercalated between two consecutive olean puffs to determine the baseline depolarization of the antenna. The EAG signals were filtered (DC to 1 kHz) with the aid of an IDAC-2 interface (Syntech), digitized on a PC, and analyzed with the EAG Pro software (version 2.0, Syntech).

### 2.6. Behavioral Bioassays

The walking response of both sexes of virgin FOB when simultaneously exposed to olean (10 and 100 µg) and charcoal-filtered pure air was evaluated in a double-choice “Y”–shaped glass olfactometer (main arm 10 cm long × 18 mm I.D., arms 8 cm long × 5 mm I.D., angle between arms 90°). A total of 21–43 individuals of both sexes and age category (<7 and >7 days old) were tested for each olean amount. The compound was loaded onto a Whatman filter paper following the same procedure described above for the EAG assays, and it was renewed after every second insect was tested. Incoming airflow for both arms was maintained at ca. 300 mL/min, and a light source placed 30 cm above the olfactometer provided a homogeneous illumination of 500 lx. All tests were conducted at 23 ± 1 °C and 50 ± 10% RH, and, before each assay, insects were individually acclimatized to room conditions for 30 min inside 15 mL Falcon^®^ tubes. The response of each FOB was assessed for 5 min; if there was no response after this time, the insect was discarded. A response was considered positive if the beetle walked at least 3 cm into one arm. After testing five consecutive insects, arms were switched over to avoid direction bias. The entire olfactometer was washed, first with soap and water, then with absolute ethanol and acetone, and left to dry in an oven at 120 °C.

### 2.7. Field Tests

Attractiveness of olean under natural conditions was evaluated in a field trial conducted from 11th June to 17th August 2021 in a private cork oak stand managed for cork production every 12–13 years (N 41.826718°, E 2.580175°, 531 m.a.s.l., Catalonia, NE Spain) and with clear symptoms of FOB infestation. Specifically, no cork harvesting had been undertaken prior to conducting the assay. The study plot (ca. 8 ha size) is almost dominated by *Q. suber* of approximately 50–150 years old, with a low presence of *Q. ilex*.

Sticky purple three-sided prism traps (100 cm long, 53 cm wide, 0.4 cm thick, peak reflectance: 430 and 750 nm; La Digital Impser, Girona, Spain) [21] made of corrugated polypropylene cardboard were deployed following a previous methodology [18]. In brief, prism traps were hung in sun-exposed areas at 1.5–2 m above the ground level by attaching them to an iron rod (2 m height), spaced at least 40 m apart to each other, and with a distance >2 m from the oak trunk. The three outer faces of the prism were coated with Tangle-Trap^®^ (The Tanglefoot Company, Grand Rapids, MI, USA).

We compared the efficacy of unbaited prism traps (*n* = 20 traps, referred to as control) against prism traps baited with olean at the release rates of ca. 0.75 mg/day (*n* = 20 traps) and 3.75 mg/day (*n* = 20 traps). For this purpose, the sticky purple traps were arranged in twenty randomized complete blocks (*n* = 3 traps per block), with a minimum distance of 40 m among blocks. Olean-releasing lures consisted of a 400 µL polyethylene tube with a snap-on cap (Beckman Coulter, Deltalab, Spain), each loaded with 140 µL of the synthetic compound. Thus, the 3.75 mg/day release rate was achieved by combining five dispensers. Lures were hung up inside the upper part of the prism trap by means of a metal wire. To ease the emission of the odor plume, six holes (2 cm diameter) were vertically drilled in the middle of each side of the prism trap. Prior to setting up the assay, the release rate of the dispenser was estimated based on the daily weight loss for two weeks under laboratory conditions. For this purpose, dispensers (*n* = 4 replicates) were hung up in a glass wind-tunnel (180 × 50 × 50 cm) at 25 ± 2 °C, 50% ± 10% RH, and exposed to a constant airflow of 19 cm/s [18]. Traps were checked every week for two months, and the FOB were counted and sexed. No reapplication of the sticky coating was undertaken after each week of collection. Females were dissected under a stereomicroscope (Leica S8 APO, Leica Microsystems, Wetzlar, Germany) to determine their reproductive status, and for comparison, virgin females (*n* = 10) emerged from net-covered *Q. suber* trunk sections were also dissected.

### 2.8. Statistical Analysis

Prior to conducting any comparison, EAG recording data were analyzed to check whether they met the assumptions of normality (Shapiro–Wilk test, sample size < 50) and homoscedasticity (Levene’s test), and therefore log-transformation was applied when necessary. Subsequent differences in absolute EAG amplitudes within a sex were analyzed by one-way analysis of variance (ANOVA), at a significance level of α = 0.05. When significant differences were detected, pairwise comparisons among olean amounts were applied (Tukey post hoc test). With regard to comparisons between sexes within a particular amount, Student’s *t*-test was applied (α = 0.05). To analyze the walking preference towards olean in the olfactometer, a chi-square test was conducted at a significance level of α = 0.05. Finally, differences among the number of FOB trap catches were analyzed by the non-parametric Kruskal–Wallis test (α = 0.05). All the statistical procedures were performed using SPSS Statistics 17.0 software (SPSS, Chicago, IL, USA).

## 3. Results

### 3.1. Headspace Solid-Phase Microextraction

**Figure 1:**
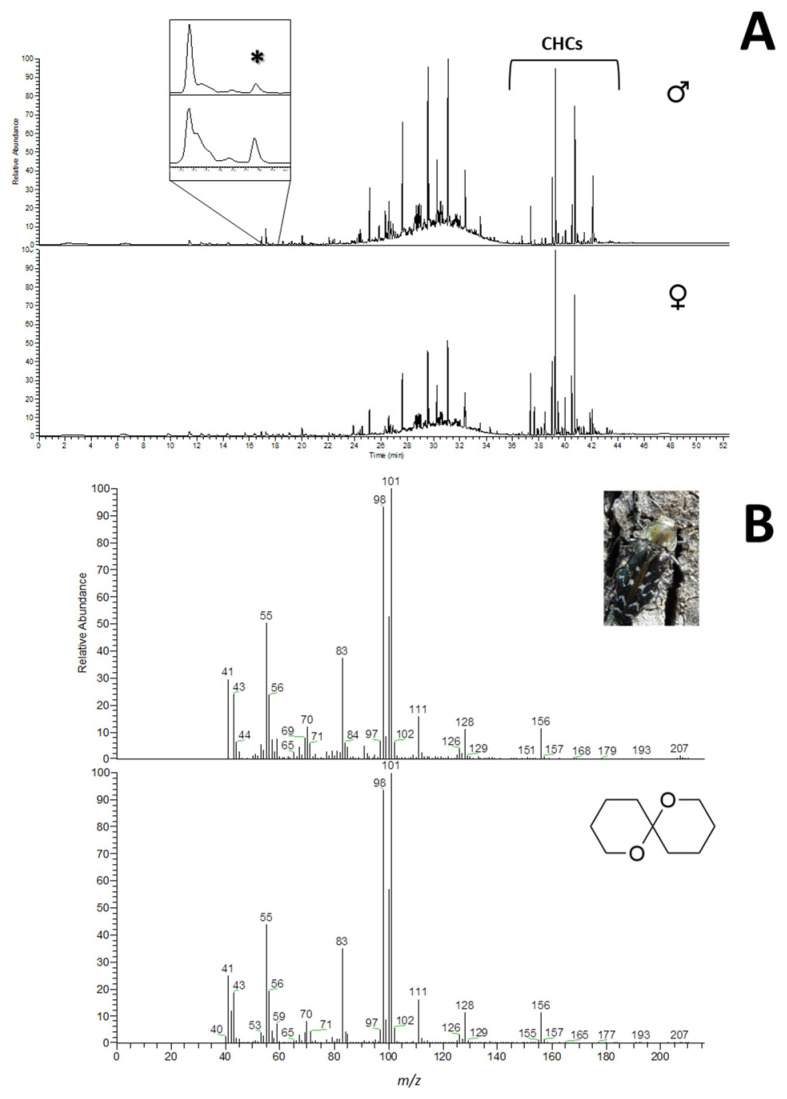
(**A**) Representative gas chromatograms obtained by HS-SPME of individually sampled *C. undatus* from both sexes, and magnified region depicting the elution time of olean (denoted with an asterisk). (**B**) Electron impact mass spectra of naturally occurring and synthetic olean. Legend: CHCs, cuticular hydrocarbons.

Volatile collection by HS-SPME of individually sampled *C. undatus* yielded gas chromatographic profiles mainly constituted by the insect long-chain cuticular hydrocarbons (for being in contact with the adsorbent fiber), and compounds derived from the own SPME fiber coating material and/or plastic contamination (Figure 1A). However, a thorough study of the volatile region of the gas chromatogram revealed the presence of a non-sex-specific compound in all the samples (males *n* = 8 and females *n* = 8) at 17.77 min (Figure 1A). Its mass spectrum showed two characteristic fragment ions at m/z 98 and 101 in high relative abundance, and the molecular ion of m/z 156, suggesting the structure of olean (Figure 1B). This particular fragmentation pattern is in agreement with those reported for structurally related compounds [22]. Further comparison of the obtained mass spectrum with those of the synthetic standard and from the NIST library confirmed the identity of the compound (Figure 1B).

### 3.2. Electroantennographic Response

**Figure 2:**
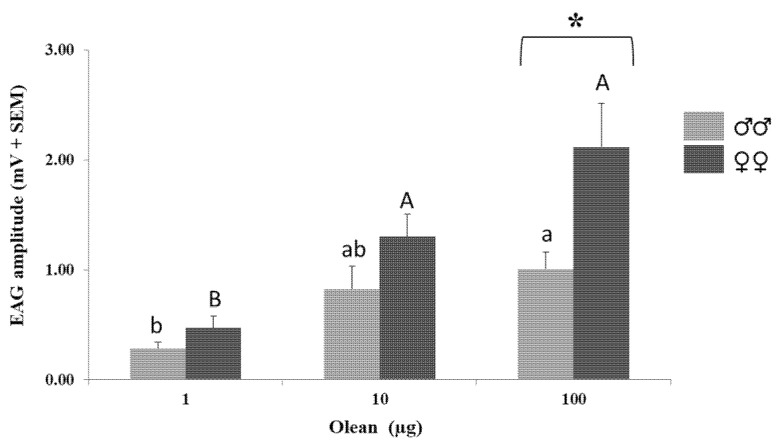
Mean electroantennographic response (mV + SEM) of *C. undatus* males (*n* = 5) and females (*n* = 10) to olean (1–100 µg). Different letters within each sex denote significant differences among olean amounts (one-way ANOVA followed by Tukey’s post hoc test, *p* < 0.05). Asterisk indicates significant differences between sexes in the response level to a concrete olean amount (Student’s *t*-test, *p* < 0.05).

Antennae of both sexes of the FOB responded to increasing amounts of olean (1, 10, and 100 µg) in a similar dose-response fashion (males, F = 5.800, df = 2, *p* = 0.016; females, F = 18.276, df = 2, *p* < 0.001). In females, the response to the lowest quantity (1 µg) was found to be statistically different to those recorded at 10 and 100 µg, whereas in males only the EAG amplitude at 100 µg differed from the response to 1 µg (Figure 2). No significant differences between sexes were detected, with the exception of 100 µg, with females displaying a higher EAG response than males (2.12 ± 0.39 vs. 1.05 ± 0.16 mV) (t = 2.388, df = 2, *p* < 0.032) (Figure 2).

### 3.3. Behavioral Assays

Overall, FOB males displayed a higher mobility percentage for either arm of the olfactometer than females, ranging from 85% to 96%, whereas the latter showed an average percentage of 65%, regardless of the age category.

**Figure 3:**
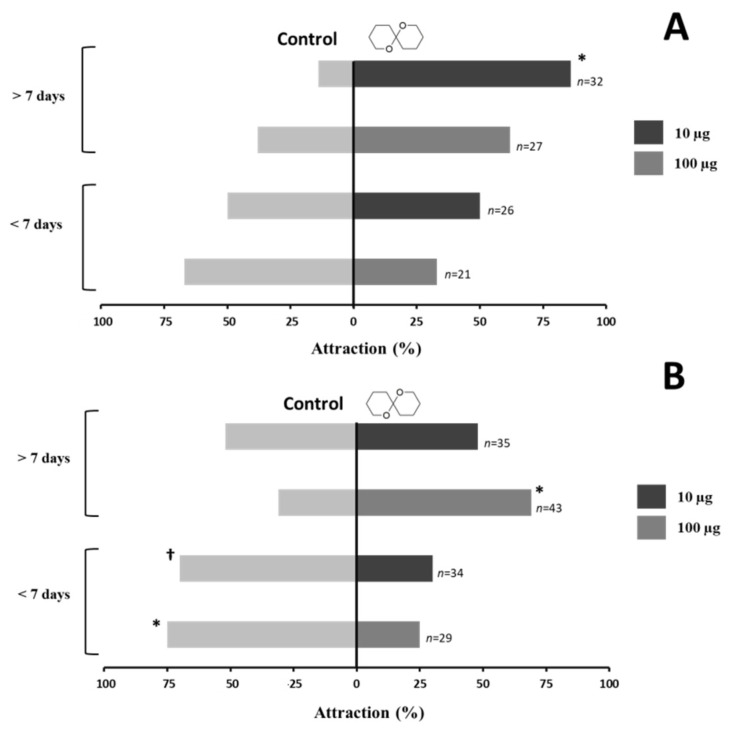
Behavioral response of *C. undatus* males (**A**) and females (**B**) of two ages (<7 and >7 days old) to olean (10 and 100 µg) in a double choice Y-shaped olfactometer. Asterisks denote a significant preference for either the olean-containing arm or the control (pure air) arm (chi-square test at α = 0.05; † = 0.06). The number beside each bar indicates the total number of individuals tested.

In terms of attractiveness to olean, 86% of males (>7 days old) made a significant choice when exposed to 10 µg of the compound (Figure 3A), whereas no significant attraction was elicited by the amount of 100 µg (62%, Figure 3A). In contrast, females (>7 days old) only showed a significant preference for olean (69%) when exposed to the highest amount (100 µg) (Figure 3B), with less than 50% of the females making a choice towards 10 µg of the chemical (Figure 3B).

Conversely, males and females younger than seven days old did not exhibit the same response pattern. In these individuals, males did not display any attraction to either amount of olean (33–50%, Figure 3A), and even an aversive behavior was evoked in females at both 10 and 100 µg, with attraction percentages of only 25–30% (Figure 3B).

### 3.4. Field Assays

**Figure 4:**
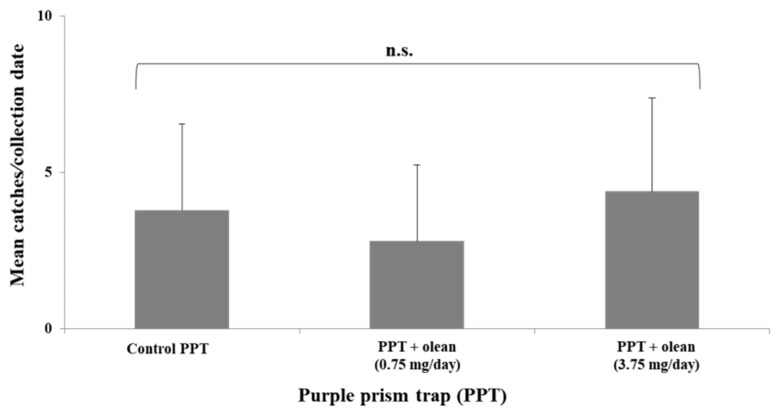
Response of *C. undatus* (mean number + SD) to olean-baited purple prism traps at two different release rates (0.75 and 3.75 mg/day, *n* = 20 traps per release rate) vs. unbaited control (*n* = 20 traps) (n.s. = not significant, Kruskal–Wallis test at α = 0.05).

Overall, a total of 55 *C. undatus* were trapped, with all of them determined to be females. First insect catches occurred on 6th July, and lasted until 4th August, when the flight activity of the insect ceased. Therefore, only catches from this five-week period were taken into consideration for data analysis. The presence of olean did not increase the number of beetles caught in comparison to unbaited control traps at either of the release rates tested (χ^2^ = 0.880, df = 2, *p* = 0.644) (Figure 4). Control traps caught an average of 3.8 ± 2.8 beetles per collection date, and the mean number of FOB found in olean-releasing traps was 2.8 ± 2.4 (olean release rate: 0.75 mg/day) and 4.4 ± 3.0 (3.75 mg/day).

**Figure 5:**
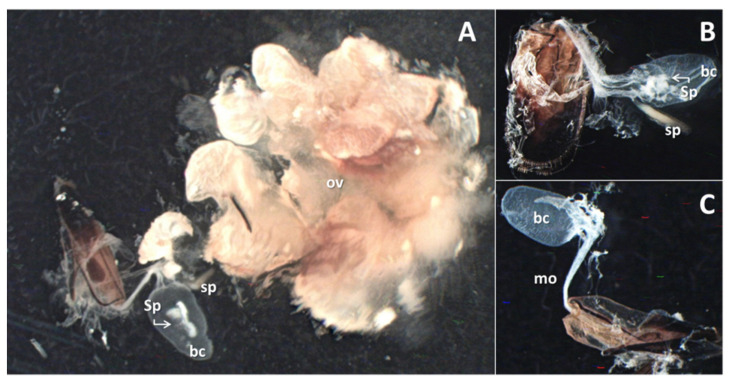
Reproductive system of a female from trap captures (**A**); close-up of the genitalia of a trapped (**B**) and virgin female (**C**). Legend: ov, ovaries; bc, bursa copulatrix; Sp, spermatophore; sp, spermatheca; mo, median oviduct.

All the females caught in traps were revealed as sexually mature and mated, characterized by the presence of well-developed ovaries (Figure 5A), and the spermatophore inside the bursa copulatrix (Figure 5A,B). In contrast, virgin females from net-covered trunk sections presented less developed ovaries and lacked the spermatophore within the bursa copulatrix (Figure 5C).

## 4. Discussion

In Buprestidae, host and mate-seeking behavior is suggested to be ruled by complex signals that integrate visual and olfactory cues at both long and short range [23,24,25,26]. In terms of olfaction, the deciphering of host kairomones has been successfully addressed in numerous studies [18,19,24,27,28,29,30], in contrast to the isolation and characterization of jewel beetle pheromones. To date, identification of pheromones in this family has been only achieved in *A. planipennis*, in which the female-released (*Z*)-3-dodecen-12-olide is considered as a short-range sex pheromone that attracts males [31,32]. In the congeneric species *Agrilus bilineatus* (Weber), the presence of females increases the attraction of males towards cages baited with host logs, suggesting the existence of female-released compound(s) with pheromonal activity [33]. In addition to volatile pheromones, scant works have aimed to the identification of contact chemical cues [29,34,35].

Here, we report for the first time the release, and the electroantennographic and behavioral activity, of olean on both sexes of the FOB. This spiroacetal compound has been previously detected only in fruit fly species (Diptera: Tephritidae), and in the stingless bee *Partamona cupira* (Smith) (Hymenoptera: Apidae) [36,37,38]. Biologically active spiroacetals have been also described as part of the pheromonal communication system of some bark beetles (Coleoptera: Curculionidae), such as (2*S*,5*S*)-chalcogran [(2*S*,5*S*)-2-ethyl-1,6-dioxaspiro[4.4]nonane] in the genus *Pityogenes* Bedel [39,40,41], and (5*S*,7*S*)-conophthorin [(5*S*,7*S*)-7-methy1-1,6-dioxaspiro[4.5]decane] in *Pityophthorus carmeli* Swaine [42], among others.

Our laboratory assays demonstrated that virgin male and female FOB more than seven days from emergence positively responded to racemic olean in behavioral tests, whereas this attraction was not mediated in insects younger than seven days old, and a repellent effect was even detected in these females. These age-related differences in response to the compound may suggest that olean has a role in the chemical communication of the insect according to its sexual maturity. After trunk emergence, new FOB may migrate to the canopy in search of fresh leaves, as other bark-boring beetles behave in maturation feeding [43,44]. Hence, young FOB would not find these chemical cues related to intraspecific communication attractive. A similar response pattern related to sexual maturity has been observed, for instance, in *A. planipennis* when responding to kairomonal cues for host selection, with immature females not being responsive to those host chemical cues [(*Z*)-3-hexen-1-ol and bark sesquiterpenes] that are highly attractive for mature conspecifics [45].

Even though the chirality of naturally-occurring olean in *C. undatus* was not elucidated, the racemate was proven to significantly attract both males and females in behavioral assays. To date, in two of the tephritid species known to use the compound as an intraspecific cue, namely *Bactrocera oleae* (Gmelin) and *Bactrocera cacuminata* (Héring), it has been determined that olean is biosynthesized as its racemic form [46,47]. In the olive fruit fly *B. oleae* both sexes produce and release the racemic mixture [47,48], with males being attracted to (*R*)-olean in laboratory and field assays, and females only responding to (*S*)-olean under laboratory conditions [47]. Although speculative, the hypothesis that *C. undatus* produces the racemic form of olean should not be ruled out, taking into consideration that the biosynthetic pathway of olean in fruit flies species is suggested to follow a general paradigm [49]. Nonetheless, the determination of the natural absolute stereochemistry would afford a more accurate picture of the activity and specificity of each isomer upon the chemical ecology of the species. In this sense, it is also worth noting that the absence of a sex-specific release of the compound contrasts with the attractiveness exerted on both sexes in olfactometric tests. In *A. planipennis*, (*Z*)-3-dodecen-12-olide is released by sexually mature females (>10 days old) [32], although Bartelt and coworkers reported its presence at trace levels in volatile collection from males [31]. Indeed, *A. planipennis* females do not respond to (*Z*)-3-dodecen-12-olide in behavioral tests under laboratory conditions, despite being electrophysiologically active [32]. Nevertheless, in our case and in spite of the behavioral activity observed in the laboratory, no enhancement in the attraction of *C. undatus* towards purple prism traps was observed when these were baited with olean at two different release rates. Interestingly, the purple prism trap was revealed to be effective in attracting only mated females, regardless of the presence of the chemical. In the same way, *A. planipennis* females found in purple traps baited with bark sesquiterpenes were determined to be more sexually developed than those in traps releasing the green leaf volatile (Z)-3-hexen-1-ol [45]. These results suggest that sexual maturity and mating status modulate the preference of females to a specific trapping system, with mated and gravid females showing a marked preference towards olfactory (bark sesquiterpenes) and visual (purple prism trap) cues that may indicate the location of possible oviposition sites. Therefore, some features of the purple prism trap, such as color properties (hue, reflectance), shape, and/or silhouette [50,51] may resemble the host to some extent, and thus lure *C. undatus* mated females seeking a suitable oviposition substrate.

As stated earlier, jewel beetles rely on visual cues to locate potential mates and hosts [52,53]. In particular, in the case of the FOB, both sexes are characterized by the presence of randomly rotated and chiral ommatidia that allow a polarized and tetrachromatic vision, based on blue, green, red, and ultraviolet [21], in a similar vein to the spectral sensitivity displayed by *A. planipennis* [52]. This suggests that the beetles are capable of perceiving a broad range of colored stimuli from their habitat, and specifically from their host and conspecifics. In this sense, we question whether the activity of olean may be related to a proper visual cue that may be relevant for virgin insects, because these have been demonstrated to be attracted to the compound in olfactometric trials. Trap color, along with trap deployment height, have been demonstrated to be critical features affecting jewel beetles catches [54,55]. For instance, purple and green have been demonstrated in several works to be very attractive colors for tree-dwelling buprestid species [56,57,58,59,60,61,62], and the performance of the same trap type may vary between the placement in the canopy or in the understory, in accordance to the activity pattern of the target species [24,55,63]. In *A. planipennis,* adults spend a considerable amount of time feeding on the canopy leaves [64], with males being more active in sunshine areas of the canopy [23,64]. Consequently, pheromone-baited green traps deployed at a height close to the canopy yield a higher number of captures [32]. Vertical stratification has been also demonstrated to be relevant in the number of catches of *Agrilus convexicollis* Redtenbacher, with more individuals trapped in green multiple-funnel traps when deployed in the canopy than in the understory [55]. This outperformance of green traps in the canopy should not be surprising, because they may be visually perceived by insects as a foliage-like stimulus [52]. To the best of our knowledge, hovering sites of *C. undatus* after emergence are still unknown. However, it is feasible that it may behave similarly to aforementioned species, with virgin individuals flying to the oak crown in search of feeding leaves and presumably mates. Within this context, assessing the role of green prism traps along with olean in the canopy may be key for determining if these stimuli are attractive to virgin FOB adults, in a similar way that purple traps result in an attractive stimulus for mated females when placed in the understory.

The efficacy of semiochemical-based management and surveillance programs in forest ecosystems has been successfully proven for different bark and wood-boring beetles [55,65,66]. However, in most cases the efficacy of this strategy is improved when sex or aggregation pheromones are co-released with host kairomones [32,67,68,69]. In the case of *A. planipennis*, the macrocyclic lactone does not exert a significant attraction when released singly, although it increases the attraction of males to (*Z*)-3-hexen-1-ol in green prism traps hung up in the canopy [32,63]. Therefore, it should not be ruled out that the activity of olean may be synergized in the presence of host volatiles with a kairomonal role. In this regard, a blend of five host green leaf volatiles, viz. (*E*)-2-hexenal, (*E*)-2-hexenol, 1-hexanol, hexyl acetate, and (*Z*)-3-hexenyl acetate, exclusively lures FOB females to purple prism traps [18]. If individually screened, their single biological activity would allow a simpler blend to be defined, thus avoiding any redundant effect [70], and additionally would contribute to determine potential host synergists for olean. Indeed, and in spite of their ubiquity, some of these plant volatiles have been previously demonstrated as strong synergists of the pheromonal activity in different insect pests [71,72,73,74].

Combined, our findings shed light on the chemical ecology of *C. undatus*, revealing for the first time the existence of a particular compound that is antennally and behaviorally active on both sexes, albeit additional investigation is still required to determine the true nature of olean as semiochemical within the intraspecific communication context. Moreover, the abovementioned arguments evidence the inconsistency between our laboratory and field results, suggesting that the development of an optimized trapping methodology for *C. undatus* should involve the integration of multiple features in a context-specific manner, including those related to the experimental set-up (trap type and color, placement, height, sun exposure, etc.), or dependent on the insect and host (i.e., population density, infestation level, etc.) [56]. Accordingly, further field trials based on a multicomponent methodology are necessary to understand the role of olean in the chemical ecology of *C. undatus*.

## Data Availability

All relevant data are included in the article, and available if necessary on request from the corresponding authors.

## References

[1] van Lierop P., Lindquist E., Sathyapala S., Franceschini G. (2015). Global forest area disturbance from fire, insect pests, diseases and severe weather events. For. Ecol. Manag..

[2] Wood D.L. (1982). The role of pheromones, kairomones, and allomones in the host selection and colonization behavior of bark beetles. Annu. Rev. Entomol..

[3] Evans H.F., Moraal L.G., Pajares J.A., Lieutier F., Day K.R., Battisti A. (2007). Biology, ecology and economic importance of buprestidae and cerambycidae. Bark and Wood Boring Insects in Living Trees in Europe, A Synthesis.

[4] Linnakoski R., Forbes K.M. (2019). Pathogens—The hidden face of forest invasions by wood-boring insect pests. Front. Plant. Sci..

[5] Wingfield M.J., Garnas J.R., Hajek A., Hurley B.P., de Beer Z.W., Taerum S.J. (2016). Novel and co-evolved associations between insects and microorganisms as drivers of forest pestilence. Biol. Invasions.

[6] Haack R.A., Benjamin D.M. (1982). The biology and ecology of the twolined chestnut borer, *Agrilus bilineatus* (Coleoptera: Buprestidae), on oaks, *Quercus* spp. in Wisconsin. Can. Entomol..

[7] Tluczek A.R., McCullough D.G., Poland T.M. (2011). Influence of host stress on emerald ash borer (Coleoptera: Buprestidae) adult density, development, and distribution in *Fraxinus pennsylvanica* trees. Environ. Entomol..

[8] Svihra P., Koehler C.S. (1993). Flatheaded borer in white alder landscape trees. J. Arboric..

[9] Barter G.W. (1957). Studies of the bronze birch borer, *Agrilus anxius* Gory, in New Brunswick. Can. Entomol..

[10] Haack R.A., Jendek E., Liu H., Marchant K.R., Petrice T.R., Poland T.M., Ye H. (2002). The emerald ash borer: A new exotic pest in North America. Newsl. Michigan Entomol. Soc..

[11] Kovacs K.F., Haight R.G., McCullough D.G., Mercader R.J., Siegert N.W., Liebhold A.M. (2010). Cost of potential emerald ash borer damage in U.S. communities, 2009–2019. Ecol. Econ..

[12] Hope E., Sun L., Mckenney D., Bogdanski B., Pedlar J., Macaulay L., Macdonald H., Lawrence K. (2020). Emerald Ash Borer, Agrilus planipennis: An. Economic Analysis of Regulations in Canada.

[13] Silk P., Mayo P., Ryall K., Roscoe L. (2019). Semiochemical and communication ecology of the emerald ash borer, *Agrilus planipennis* (Coleoptera: Buprestidae). Insects.

[14] Gallardo A., Jiménez A., Antonietty C.A., Villagrán M., Ocete M.E., Soria F.J. (2012). Forecasting infestation by *Coraebus undatus* (Coleoptera, Buprestidae) in cork oak forests. Int. J. Pest. Manag..

[15] Codina A. (1926). Nota sobre el corc del suro *Coraebus undatus* F. (Col. Buprestidae). Bull. Inst. Cat. Hist. Nat..

[16] Wachtendorf W. (1955). Beiträge zur Kenntnis der Eichenprachtkäfer *Agrilus biguttatus* Fabr. und *Coraebus undatus* Fabr. (Col. Bupr.). Z. für Angew. Entomol..

[17] Soria F.J., Villagrán M., Ocete M.E. (1992). Estudios poblacionales sobre *Coroebus undatus* (Fabricius) (Coleoptera, Buprestidae) en alcornocales de Andalucía occidental. II: Aspectos ecológicos de la larva. Bol. San. Veg. Plagas.

[18] Fürstenau B., Quero C., Riba J.M., Rosell G., Guerrero A. (2015). Field trapping of the flathead oak borer *Coroebus undatus* (Coleoptera: Buprestidae) with different traps and volatile lures. Insect Sci..

[19] Fürstenau B., Rosell G., Guerrero A., Quero C. (2012). Electrophysiological and behavioral responses of the black-banded oak borer, *Coroebus florentinus*, to conspecific and host-plant volatiles. J. Chem. Ecol..

[20] Kubáň V., Majer K., Kolibáč J. (2000). Classification of the tribe Coraebini Bedel, 1921 (Coleoptera, Buprestidae, Agrilinae). Acta Mus. Morav. Sci. Biol..

[21] Meglič A., Ilić M., Quero C., Arikawa K., Belušič G. (2020). Two chiral types of randomly rotated ommatidia are distributed across the retina of the flathead oak borer *Coraebus undatus* (Coleoptera: Buprestidae). J. Exp. Biol..

[22] Francke W., Hindorf G., Reith W. (1979). Mass-spectrometric fragmentation of alkyl-1,6-dioxaspiro[4.5]decanes. Naturwissenschaften.

[23] Lelito J.P., Fraser I., Mastro V.C., Tumlinson J.H., Böröczky K., Baker T.C. (2007). Visually mediated “paratrooper copulations” in the mating behavior of *Agrilus planipennis* (Coleoptera: Buprestidae), a highly destructive invasive pest of North American ash trees. J. Insect Behav..

[24] Crook D.J., Khrimian A., Francese J.A., Fraser I., Poland T.M., Sawyer A.J., Mastro V.C. (2008). Development of a host-based semiochemical lure for trapping emerald ash borer *Agrilus planipennis* (Coleoptera: Buprestidae). Environ. Entomol..

[25] Pureswaran D.S., Poland T.M. (2009). The role of olfactory cues in short-range mate finding by the emerald ash borer, *Agrilus planipennis* Fairmaire (Coleoptera: Buprestidae). J. Insect Behav..

[26] Lelito J.P., Domingue M.J., Fraser I., Mastro V.C., Tumlinson J.H., Baker T.C. (2011). Field investigation of mating behaviour of *Agrilus cyanescens* and *Agrilus subcinctus*. Can. Entomol..

[27] Vuts J., Woodcock C.M., Sumner M.E., Caulfield J.C., Reed K., Inward D.J., Leather S.R., Pickett J.A., Birkett M.A., Denman S. (2016). Responses of the two-spotted oak buprestid, *Agrilus biguttatus* (Coleoptera: Buprestidae), to host tree volatiles. Pest. Manag. Sci..

[28] De Groot P., Grant G.G., Poland T.M., Scharbach R., Buchan L., Nott R.W., MacDonald L., Pitt D. (2008). Electrophysiological response and attraction of emerald ash borer to green leaf volatiles (GLVs) emitted by host foliage. J. Chem. Ecol..

[29] Bari G., Scala A., Garzone V., Salvia R., Yalcin C., Vernile P., Aresta A.M., Facini O., Baraldi R., Bufo S.A. (2019). Chemical ecology of *Capnodis tenebrionis* (L.) (Coleoptera: Buprestidae): Behavioral and biochemical strategies for intraspecific and host interactions. Front. Physiol..

[30] Coleman T.W., Chen Y., Graves A.D., Hishinuma S.M., Grulke N.E., Flint M.L., Seybold S.L. (2014). Developing monitoring techniques for the invasive goldspotted oak borer (Coleoptera: Buprestidae) in California. Environ. Entomol..

[31] Bartelt R.J., Cossé A.A., Zilkowski B.W., Fraser I. (2007). Antennally active macrolide from the emerald ash borer *Agrilus planipennis* emitted predominantly by females. J. Chem. Ecol..

[32] Silk P.J., Ryall K., Mayo P., Lemay M.A., Grant G., Crook D., Cossé A., Fraser I., Sweeney J.D., Lyons D.B. (2011). Evidence for a volatile pheromone in *Agrilus planipennis* Fairmaire (Coleoptera: Buprestidae) that increases attraction to a host foliar volatile. Environ. Entomol..

[33] Dunn J.P., Potter D.A. (1988). Evidence for sexual attraction by the twolined chestnut borer *Agrilus bilineatus* (Weber) (Coleoptera: Buprestidae). Can. Entomol..

[34] Lelito J.P., Böröczky K., Jones T.H., Fraser I., Mastro V.C., Tumlinson J.H., Baker T.C. (2009). Behavioral evidence for a contact sex pheromone component of the emerald ash borer, *Agrilus planipennis* Fairmaire. J. Chem. Ecol..

[35] Silk P.J., Ryall K., Barry Lyons D., Sweeney J., Wu J. (2009). A contact sex pheromone component of the emerald ash borer *Agrilus planipennis* Fairmaire (Coleoptera: Buprestidae). Naturwissenschaften.

[36] Francke W., Kitching W. (2005). Spiroacetals in Insects. Curr. Org. Chem..

[37] Noushini S., Park S.J., Jamie I., Jamie J., Taylor P. (2021). Rectal gland exudates and emissions of *Bactrocera bryoniae*: Chemical identification, electrophysiological and pheromonal functions. Chemoecology.

[38] Helms A.M., De Moraes C.M., Tröger A., Alborn H.T., Francke W., Tooker J.F., Mescher M.C. (2017). Identification of an insect-produced olfactory cue that primes plant defenses. Nat. Commun..

[39] Francke W., Heemann V., Gerken B., Renwick J.A.A., Vité J.P. (1977). 2-Ethyl-1,6-dioxaspiro[4.4]nonane, principal aggregation pheromone of *Pityogenes chalcographus* (L.). Naturwissenschaften.

[40] Byers J.A., Birgersson G., Francke W. (2013). Aggregation pheromones of bark beetles, *Pityogenes quadridens* and *P. bidentatus*, colonizing Scotch pine: Olfactory avoidance of interspecific mating and competition. Chemoecology.

[41] Birgersson G., Dalusky M.J., Berisford C.W. (2000). Identification of an aggregation pheromone for *Pityogenes hopkinsi* (Coleoptera: Scolytidae). Can. Entomol..

[42] Dallara P.L., Seybold S.J., Meyer H., Tolasch T., Francke W., Wood D.L. (2000). Semiochemicals from three species of *Pityophthorus* (Coleoptera: Scolytidae): Identification and field response. Can. Entomol..

[43] Långström B. (1983). Life Cycles and Shoot-Feeding of the Pine Shoot Beetles.

[44] Haack R.A., Wang Q. (2017). Feeding biology of cerambycids. Cerambycidae of the World; Biology and Pest Management.

[45] Ryall K.L., Dutkiewicz D., Silk P.J., Antunes P.M., Ochoa I. (2013). Ovarian development of *Agrilus planipennis*: Effects of age and mating status and influence on attraction to host volatiles. Entomol. Exp. Appl..

[46] Krohn S., Fletcher M.T., Kitching W., Drew R.A.I., Moore C.J., Francke W. (1991). Chemistry of fruit flies: Nature of glandular secretion and volatile emission of *Bactrocera* (*Bactrocera*) *cacuminatus* (Héring). J. Chem. Ecol..

[47] Haniotakis G., Francke W., Mori K., Redlich H., Schurig V. (1986). Sex-specific activity of (*R*)-(-)- and (*S*)- (+)-1,7-dioxaspiro[5.5]undecane, the major pheromone of *Dacus oleae*. J. Chem. Ecol..

[48] Levi-Zada A., Nestel D., Fefer D., Nemni-Lavy E., Deloya-Kahane I., David M. (2012). Analyzing diurnal and age-related pheromone emission of the olive fruit fly, *Bactrocera oleae* by sequential SPME-GCMS analysis. J. Chem. Ecol..

[49] Singh A.A., Rowley J.A., Schwartz B.D., Kitching W., De Voss J.J. (2014). Oxidative carbon–carbon bond cleavage is a key step in spiroacetal biosynthesis in the fruit fly *Bactrocera cacuminata*. J. Org. Chem..

[50] Campbell S.A., Borden J.H. (2005). Bark reflectance spectra of conifers and angiosperms: Implications for host discrimination by coniferophagous bark and timber beetles. Can. Entomol..

[51] Strom B.L., Goyer R.A. (2001). Effect of silhouette color on trap catches of *Dendroctonus frontalis* (Coleoptera: Scolytidae). Ann. Entomol. Soc. Am..

[52] Crook D.J., Francese J.A., Zylstra K.E., Fraser I., Sawyer A.J., Bartels D.W., Lance D.R., Mastro V.C. (2009). Laboratory and field response of the emerald ash borer (Coleoptera: Buprestidae), to selected regions of the electromagnetic spectrum. J. Econ. Entomol..

[53] Domingue M.J., Lelito J.P., Myrick A.J., Csóka G., Szőcs L., Imrei Z., Baker T.C. (2016). Differences in spectral selectivity between stages of visually-guided mating approaches in a buprestid beetle. J. Exp. Biol..

[54] Imrei Z., Lohonyai Z., Csóka G., Muskovits J., Szanyi S., Vétek G., Fail J., Tóth M., Domingue M.J. (2020). Improving trapping methods for buprestid beetles to enhance monitoring of native and invasive species. For. An. Int. J. For. Res..

[55] Rassati D., Marini L., Marchioro M., Rapuzzi P., Magnani G., Poloni R., Di Giovanni F., Mayo P., Sweeney J. (2019). Developing trapping protocols for wood-boring beetles associated with broadleaf trees. J. Pest. Sci..

[56] Poland T.M., Petrice T.R., Ciaramitaro T.M. (2019). Trap Designs, colors, and lures for emerald ash borer detection. Front. For. Glob. Chang..

[57] Silk P.J., Ryall K.L., Grant G., Roscoe L.E., Mayo P., Williams M., LeClair G., Kimoto T., Williams D., Rutledge C. (2019). Tree girdling and host tree volatiles provides a useful trap for bronze birch borer *Agrilus anxius* Gory (Coleoptera: Buprestidae). For. An. Int. J. For. Res..

[58] Cavaletto G., Faccoli M., Marini L., Spaethe J., Magnani G., Rassati D. (2020). Effect of trap color on captures of bark- and wood-boring beetles (Coleoptera; Buprestidae and Scolytinae) and associated predators. Insects.

[59] Petrice T.R., Haack R.A., Poland T.M. (2013). Attraction of *Agrilus planipennis* (Coleoptera: Buprestidae) and other buprestids to sticky traps of various colors and shapes. Great Lakes Entomol..

[60] Brown N., Jeger M., Kirk S., Williams D., Xu X., Pautasso M., Denman S. (2017). Acute oak decline and *Agrilus biguttatus*: The co-occurrence of stem bleeding and D-shaped emergence holes in Great Britain. Forests.

[61] Francese J.A., Crook D.J., Fraser I., Lance D.R., Sawyer A.J., Mastro V.C. (2010). Optimization of trap color for emerald ash borer (Coleoptera: Buprestidae). J. Econ. Entomol..

[62] Domingue M.J., Imrei Z., Lelito J.P., Muskovits J., Janik G., Csóka G., Mastro V.C., Baker T.C. (2013). Trapping of European buprestid beetles in oak forests using visual and olfactory cues. Entomol. Exp. Appl..

[63] Ryall K.L., Silk P.J., Mayo P., Crook D., Khrimian A., Cossé A.A., Sweeney J., Scarr T. (2012). Attraction of *Agrilus planipennis* (Coleoptera: Buprestidae) to a volatile pheromone: Effects of release rate, host volatile, and trap placement. Environ. Entomol..

[64] Rodriguez-Saona C.R., Miller J.R., Poland T.M., Kuhn T.M., Otis G.W., Turk T., Ward D.L. (2007). Behaviors of adult *Agrilus planipennis* (Coleoptera: Buprestidae). Great Lakes Entomol..

[65] Schlyter F., Zhang Q.-H., Liu G.-T., Ji L.-Z. (2001). A successful case of pheromone mass trapping of the bark beetle *Ips duplicatus* in a forest island, analysed by 20-year time-series data. Integr. Pest. Manag. Rev..

[66] Seybold S.J., Bentz B.J., Fettig C.J., Lundquist J.E., Progar R.A., Gillette N.E. (2018). Management of western North American bark beetles with semiochemicals. Annu. Rev. Entomol..

[67] Pajares J.A., Álvarez G., Hall D.R., Ibarra N., Hoch G., Halbig P., Cocoş D., Johansson H., Schroeder M. (2017). Attractants for management of the pine sawyer beetle *Monochamus sutor*, a potential vector of *Bursaphelenchus xylophilus*. J. Appl. Entomol..

[68] Álvarez G., Gallego D., Hall D.R., Jactel H., Pajares J.A. (2016). Combining pheromone and kairomones for effective trapping of the pine sawyer beetle *Monochamus galloprovincialis*. J. Appl. Entomol..

[69] Chen G., Song Y., Wang P., Chen J., Zhang Z., Wang S., Huang X., Zhang Q.-H. (2015). Semiochemistry of *Dendroctonus armandi* Tsai and Li (Coleoptera: Curculionidae: Scolytinae): Both female-produced aggregation pheromone and host tree kairomone are critically important. Chemoecology.

[70] Bruce T.J.A., Pickett J.A. (2011). Perception of plant volatile blends by herbivorous insects—Finding the right mix. Phytochemistry.

[71] Reddy G.V.P., Guerrero A. (2000). Behavioral responses of the diamondback moth, *Plutella xylostella*, to green leaf volatiles of *Brassica oleracea* subsp. capitata. J. Agric. Food Chem..

[72] Dickens J.C., Jang E.B., Light D.M., Alford A.R. (1990). Enhancement of insect pheromone responses by green leaf volatiles. Naturwissenschaften.

[73] Savoie A., Borden J.H., Pierce H.D., Gries R., Gries G. (1998). Aggregation pheromone of *Pityogenes knechteli* and semiochemical-based interactions with three other bark beetles. J. Chem. Ecol..

[74] Deglow E.K., Borden J.H. (1998). Green leaf volatiles disrupt and enhance response to aggregation pheromones by the ambrosia beetle, *Gnathotrichus sulcatus* (Coleoptera: Scolytidae). Can. J. For. Res..

